# Identification of immune-related prognostic biomarkers in lung squamous cell carcinoma microenvironment

**DOI:** 10.3389/fimmu.2025.1724319

**Published:** 2026-01-05

**Authors:** Zhihao Wang, Zhengsheng Wu

**Affiliations:** 1 Department of Pathology, School of Basic Medical Sciences, Anhui Medical University, Hefei, Anhui, China; 2 Department of Pathology, The First Affiliated Hospital of Anhui Medical University, Hefei, Anhui, China

**Keywords:** immune-related genes, lung squamous cell carcinoma, NR1D2, prognosis, tumor microenvironment

## Abstract

**Background:**

Lung squamous cell carcinoma (LUSC) is a leading cause of cancer-related mortality. Although immunotherapy has recently demonstrated clinical benefits, the biological roles of immune-related genes (IRGs) in LUSC remain insufficiently understood.

**Methods:**

In this study, transcriptomic and clinical data from 493 LUSC patients were obtained from The Cancer Genome Atlas (TCGA). IRGs were identified through weighted gene co-expression network analysis, followed by univariate Cox regression and least absolute shrinkage and selection operator (LASSO) regression to screen for prognostic genes and establish a risk prediction model. The model’s predictive performance was validated, and the immune landscape associated with distinct risk subgroups was systematically characterized. Expression patterns and clinical significance of the signature genes were further investigated using bioinformatics analysis, quantitative real-time PCR, Western blotting, and immunohistochemistry.

**Results:**

A total of 55 differentially expressed IRGs were identified, among which 8 genes (*PSMD1, ANGPTL4, LTBP3, MIF, NFATC3, NR1D2, PLXNB3*, and *SP1*) demonstrated significant prognostic value. A prognostic signature based on these 8 IRGs was established that stratified patients into high- and low-risk groups with distinct survival outcomes, immune landscapes, and enriched pathways. As one of the constituent genes of the risk model, *NR1D2* was found to be downregulated in LUSC and associated with poor prognosis. Functional assays indicated that *NR1D2* facilitated malignant progression by regulating macrophage polarization and enhancing tumor cell migration.

**Conclusion:**

This study establishes a novel IRGs-based prognostic signature with potential utility for risk stratification and individualized immunotherapeutic strategies in LUSC. Furthermore, it also provides valuable insights into the role of *NR1D2* in clinical outcomes.

## 1 Introduction

Lung squamous cell carcinoma (LUSC) remains a major public health challenge, being one of the predominant subtypes of non-small cell lung cancer (NSCLC) and accounting for approximately 20-30% of all lung cancer cases. Despite advances in diagnostic and therapeutic strategies, LUSC continues to have a poor prognosis, with a five-year survival rate remaining dismally low (1). Unlike lung adenocarcinoma, which has benefited from the development of targeted therapies such as EGFR and ALK inhibitors (2, 3), LUSC lacks well-defined molecular targets, and treatment options are often limited to platinum-based chemotherapy and immune checkpoint inhibitors (ICIs). The advent of immunotherapy, particularly programmed death-ligand 1 (PD-L1) and programmed cell death protein 1 (PD-1) inhibitors, has significantly improved the clinical outcomes of some LUSC patients (4, 5). However, only a subset of patients demonstrates a durable response to these treatments, highlighting the need for predictive biomarkers that can guide patient stratification and personalized therapeutic strategies.

The tumor microenvironment (TME) plays a crucial role in shaping tumor progression and treatment responses in LUSC (6). Tumor-infiltrating immune cells, inflammatory mediators, and immune-related signaling pathways collectively determine the immunogenicity of tumors, influencing their susceptibility to immunotherapy (7, 8). In this context, immune-related genes (IRGs) have emerged as key regulators of tumor-immune interactions, playing pivotal roles in immune evasion, inflammation, and tumor progression. To date, emerging studies have reported methods for predicting the survival and prognosis of patients based on gene expression. So far, many cancers have constructed models through IRGs to predict prognosis, such as colorectal cancer (9), pan cancer (10), and breast cancer (11). However, their potential as predictive biomarkers and therapeutic targets in LUSC remains largely unexplored. Identifying IRGs that are associated with patient prognosis and understanding their roles in tumor immunity may provide valuable insights into the development of novel immunotherapeutic approaches and precision medicine strategies.

In this study, we aimed to construct an IRG prognostic signature for LUSC and investigate its potential implications in immunotherapy response and TME modulation. By identifying high-risk patients who may benefit from more immunotherapeutic interventions or combination therapies, our prognostic model provides a novel tool for precision oncology, enabling early risk prediction and personalized immunotherapeutic strategies.

## 2 Materials and methods

### 2.1 Data collection and processing

Transcriptomic RNA-FPKM data and corresponding clinical data for LUSC patients were obtained from The Cancer Genome Atlas (TCGA) database (https://portal.gdc.cancer.gov/). A total of 551 samples were mined, comprising 51 normal tissue samples and 500 LUSC tumor samples. Only primary LUSC tumor samples with complete survival follow-up information were retained. FPKM data were log2-transformed and normalized across samples.

### 2.2 Immune-related gene selection and weighted gene co-expression network analysis

IRGs were obtained from the ImmPort database (https://www.immport.org/). The expression matrix of these IRGs was then extracted from the transcriptional information. Weighted Gene Co-expression Network Analysis (WGCNA) was performed to identify co-expressed IRG modules. Hierarchical clustering was first used to detect and remove outlier samples. The pickSoftThreshold function was employed to determine the appropriate soft-threshold power (β) based on scale-free topology analysis. A power of β = 2 was selected because it was the lowest value achieving an acceptable scale-free topology fit while maintaining adequate mean connectivity. Module merging was performed using a cut height of 0.3 (eigengene correlation = 0.7), and network quality was evaluated using the scale-free topology fit index, mean connectivity, and module independence. This power was used to construct the adjacency matrix and subsequently the Topological Overlap Matrix (TOM), which quantifies gene interconnectivity. Hierarchical clustering based on TOM dissimilarity (1-TOM) was performed using the hclust function. Dynamic module detection was then carried out using the cutree Dynamic function, defining modules as groups of genes with high topological overlap (minimum module size = 10). Modules exhibiting high correlation coefficients (calculated using the module Eigengenes function) were merged (cutting height = 0.3). The correlation between each module’s eigengene (representative expression profile) and clinical prognostic features was assessed using Pearson correlation to identify significant modules for further analysis. Modules demonstrating significant correlations with clinical prognosis were identified for further investigation.

### 2.3 Identification of prognostic IRGs

Genes and their expression profiles were extracted from the prognostic modules identified by WGCNA. Univariate Cox regression analysis was then performed on all genes within these prognostic modules using the survival R package. IRGs demonstrating a significant association with patient survival (Benjamini-Hochberg adjusted *P*-value (FDR) < 0.05) were selected as candidate prognostic genes for subsequent analysis.

### 2.4 Construction and validation of an IRG prognostic signature

Lasso regression was applied to screen out the most significant risk factors to select the most significant prognostic biomarkers and construct the survival-predicting classifier for LUSC. A risk score (RS) for each patient was calculated using the formula:


$$Risk\;score=\sum genes_{cox\;coefficient}\times genes_{\mathrm{expression}\;level}$$

Patients were stratified into low-risk and high-risk groups based on the cohort-specific median risk score. The entire cohort of LUSC patients was randomly allocated into training and validation sets using a 1:1 simple randomization procedure. To ensure methodological reproducibility, the randomization was implemented using the *sample()* function in R (version 4.0.1) with a fixed seed (seed = 12345). This simple randomization approach was selected based on its appropriateness for our cohort size and study design, effectively minimizing selection bias while maintaining statistical power. Subsequently, the predictive accuracy of the signature was assessed using Receiver Operating Characteristic (ROC) curve analysis and calculating the Area Under the Curve (AUC) in both training and validation cohorts. Calibration curves were plotted to evaluate the agreement between the predicted survival probabilities from the signature and the actual observed outcomes in both cohorts. Kaplan-Meier survival analysis with the log-rank test was performed to compare survival outcomes between low-risk and high-risk groups in both the training and validation cohorts.

### 2.5 Differential gene expression and pathway analysis

Differentially expressed genes (DEGs) between predefined risk groups were identified using edgeR with significance thresholds of |log_2_FC| ≥1 and adjusted P < 0.05. Significant DEGs were visualized through volcano plots. Functional enrichment analysis of DEGs was subsequently performed via KOBAS 3.0, interrogating KEGG, Reactome, and PANTHER pathway databases with statistical significance set at FDR-adjusted P<0.05.

### 2.6 Characterization of the tumor immune microenvironment

Immune landscape was characterized via CIBERSORT, estimating fractions of 22 immune cell types (sample-level P < 0.05). Wilcoxon rank-sum tests identified differentially infiltrated cells between risk groups (FDR<0.05). Tumor microenvironment components were scored using ESTIMATE, while Spearman correlation linked signature genes to immune cell alterations.

### 2.7 Patients and tissue samples

A total of 45 paraffin-embedded LUSC tissue samples were obtained from patients who underwent surgical resection at the First Affiliated Hospital of Anhui Medical University (Hefei, Anhui, China) between 2020 and 2024. These samples were used for immunohistochemistry (IHC) analysis. In addition, 15 fresh LUSC tissue samples were collected from patients during the same period and immediately snap-frozen in liquid nitrogen. Total RNA and protein were subsequently extracted from the fresh tissues for quantitative real-time PCR (qRT-PCR) and Western blot (WB) analysis, respectively.

All patients were pathologically diagnosed with LUSC according to the World Health Organization (WHO) classification. Inclusion criteria included: (1) availability of complete clinical and pathological data; (2) no prior history of radiotherapy, chemotherapy, or targeted therapy before surgery; and (3) sufficient tumor tissue for analysis. Patients with incomplete clinical information, prior treatment, or coexisting malignancies were excluded. Clinicopathological characteristics such as age, sex, tumor stage, lymph node status, and histological grade were retrieved from medical records and independently reviewed by two pathologists.

Written informed consent was obtained from all patients before sample collection. The study was conducted in accordance with the Declaration of Helsinki and was approved by the Institutional Review Board of the First Affiliated Hospital of Anhui Medical University (approval number: [2024284]).

### 2.8 Cell lines and cell culture

The human LUSC cell lines Calu-1 and NCI-H520, and the human monocytic cell line THP-1 were obtained from the American Type Culture Collection (ATCC). Calu-1 and NCI-H520 cells were cultured in DMEM, supplemented with 10% FBS, 1% penicillin-streptomycin, under standard conditions (37°C, 5% CO_2_). THP-1 cells were maintained in suspension culture in RPMI-1640 + 10% FBS. All cell lines were authenticated by short tandem repeat (STR) profiling and routinely tested for mycoplasma contamination. Cells were not passaged more than 20 times before use.

### 2.9 Immunohistochemistry

45 paraffin-embedded LUSC tissues, containing 45 tumors and 45 paracancer tissues, were subjected to immunohistochemical staining using specific primary antibodies NR1D2 (1:200, Abmart). Protein expression was semiquantitatively assessed by two independent pathologists under a double-blind manner according to the staining intensity and the percentage of positive cells: staining intensity (0=negative; 1=pale yellow; 2=medium brown; 3=dark brown) and positive cell percentage (0 = 0%; 1 = 5-25%; 2 = 26-50%; 3 = 51-75%; 4>75%). The composite score (intensity + percentage) determined expression groups: low expression (<6) versus high expression (≥6), with final scores derived from the average of five randomly selected 400× high-power fields per section. Samples were classified into negative or positive expression groups accordingly.

### 2.10 Quantitative real-time polymerase chain reaction

Total RNA was isolated from fresh lung squamous cell carcinoma tissues using TRIzol reagent (Invitrogen, Waltham, USA) and reverse-transcribed into cDNA with the PrimeScript RT kit (Takara, Japan). qRT-PCR was performed using PCR arrays (TransGen, Beijing, China) according to the manufacturer’s instructions. GAPDH served as the internal control. The primer sequences for *NR1D2* were forward 5′-TTTAGTGGCATGGTTCTACTGTG-3′ and reverse 5′-AGCCTTCGCAAGCATGAACT-3′; for GAPDH, forward 5′-CGCGCCCCCGGTTTCTA-3′ and reverse 5′-GGCTCGGCTGGCGAC-3′. Relative gene expression was calculated using the 2−ΔΔCt method.

### 2.11 Western blotting

Total proteins from LUSC tissues and cells were extracted with RIPA buffer (Sigma-Aldrich, USA), quantified by BCA assay, and separated by SDS-PAGE. Proteins were transferred to PVDF membranes, blocked with 5% non-fat milk, and incubated overnight at 4°C with NR1D2(1:1000, Abmart) and GAPDH antibodies (1:1000; Cell Signaling Technology). After incubation with HRP-conjugated secondary antibodies, protein bands were detected using the enhanced chemiluminescence (ECL, WBKLS0500, Millipore, USA) and quantified with ImageJ software.

### 2.12 Transfection of cell lines

*NR1D2*-specific siRNA and negative control siRNA for human were purchased from GenePharma Co., Ltd. (Shanghai, China). Cells were transfected with siRNAs using RNAi-MAX Lipofectamine (Invitrogen), according to the manufacturer’s protocol. After 72 h, *NR1D2* knockdown efficiency was assessed by qRT-PCR and Western blot analysis. siRNA sequences: si*NR1D2*#1: 5′-CCAAUGAGUAAGUCUCCAUAU-3′; si*NR1D2*#2: 5′-CCAGUACAAGAAGUGCCUGAA-3′.

### 2.13 Wound healing assay

In the wound-healing assay, 24-well plates were used to seed cells. Cells were scratched perpendicular to the previously painted line using a sterile tip. After imaging the scratch wounds with a light microscope, cell migration was measured at time points of 0 and 24 h and quantified using ImageJ software.

### 2.14 Transwell assay

Transwell assays were performed using 24-well chambers (8-μm pore; Corning) to assess the cellular migration and invasion capabilities of Calu1 and NCI-H520 cell lines. For migration assays, 5 × 10^4^ cells in serum-free medium (0.1% BSA) were seeded into uncoated upper chambers. For invasion assays, chambers were pre-coated with Matrigel (BD Biosciences; 1:8 dilution, polymerized at 4°C). Both assays employed 600 μL of 10% FBS medium in lower chambers as a chemoattractant. After 24h incubation (37°C, 5% CO_2_), migrated/invaded cells were fixed with 4% paraformaldehyde (15 min), stained with 0.1% crystal violet (20 min), and quantified by counting five random 200× fields per membrane using ImageJ (NIH), with ≥3 biological replicates per condition.

### 2.15 Flow cytometry assay

To model paracrine signaling, a Transwell-based co-culture system was established. First, THP-1 monocytes were differentiated into M2 macrophages. Briefly, THP-1 cells were treated with 100 ng/mL phorbol 12-myristate 13-acetate (PMA) for 24 hours to induce macrophage differentiation, followed by polarization with 20 ng/mL IL-4 and 20 ng/mL IL-13 for 48 hours to obtain the M2 phenotype. These THP-1-derived M2 macrophages were then seeded in the lower chamber of a 6-well plate at a density of 5×10^5 cells per well. Concurrently, LUSC cell lines (Calu1 and NCI-H520) were pre-treated for 24 hours with conditioned supernatant from control (CS) or si*NR1D2*-transfected (si*NR1D2*CS) cultures. Subsequently, the pre-treated tumor cells were harvested, resuspended in 10% DMEM at a density of 5×10^6 cells/mL, and 100 µL of the cell suspension was added to the upper chamber of a Transwell insert (0.4 µm pore size), which was placed above the macrophage layer. This setup resulted in a final effector-to-target ratio of 1:1. Cells were co-cultured for 48 hours. After co-culture, THP-1 cells from the lower chamber were harvested for flow cytometry analysis. Approximately 1×10^6 cells were pelleted by centrifugation at 2000 rpm for 5 minutes, washed twice with PBS, and then stained in the dark for 30 minutes at room temperature with fluorescently conjugated antibodies against CD206 (BioLegend,321104) and CD163 (BioLegend, 326509). The stained cells were analyzed using a flow cytometer (BD Biosciences, USA) to quantify the expression of these M2 macrophage markers.

### 2.16 Enzyme-linked immunosorbent assay

Levels of IL-10 and TGF-β1 in culture supernatants were measured using commercially available ELISA kits (IL-10: D1000B; TGF-β1: DB100C; Quantikine, R&D Systems, Minneapolis, USA) according to the manufacturer’s instructions. Each sample was tested in triplicate, and experiments were repeated independently at least once.

### 2.17 Statistical analysis

**Figure 1:**
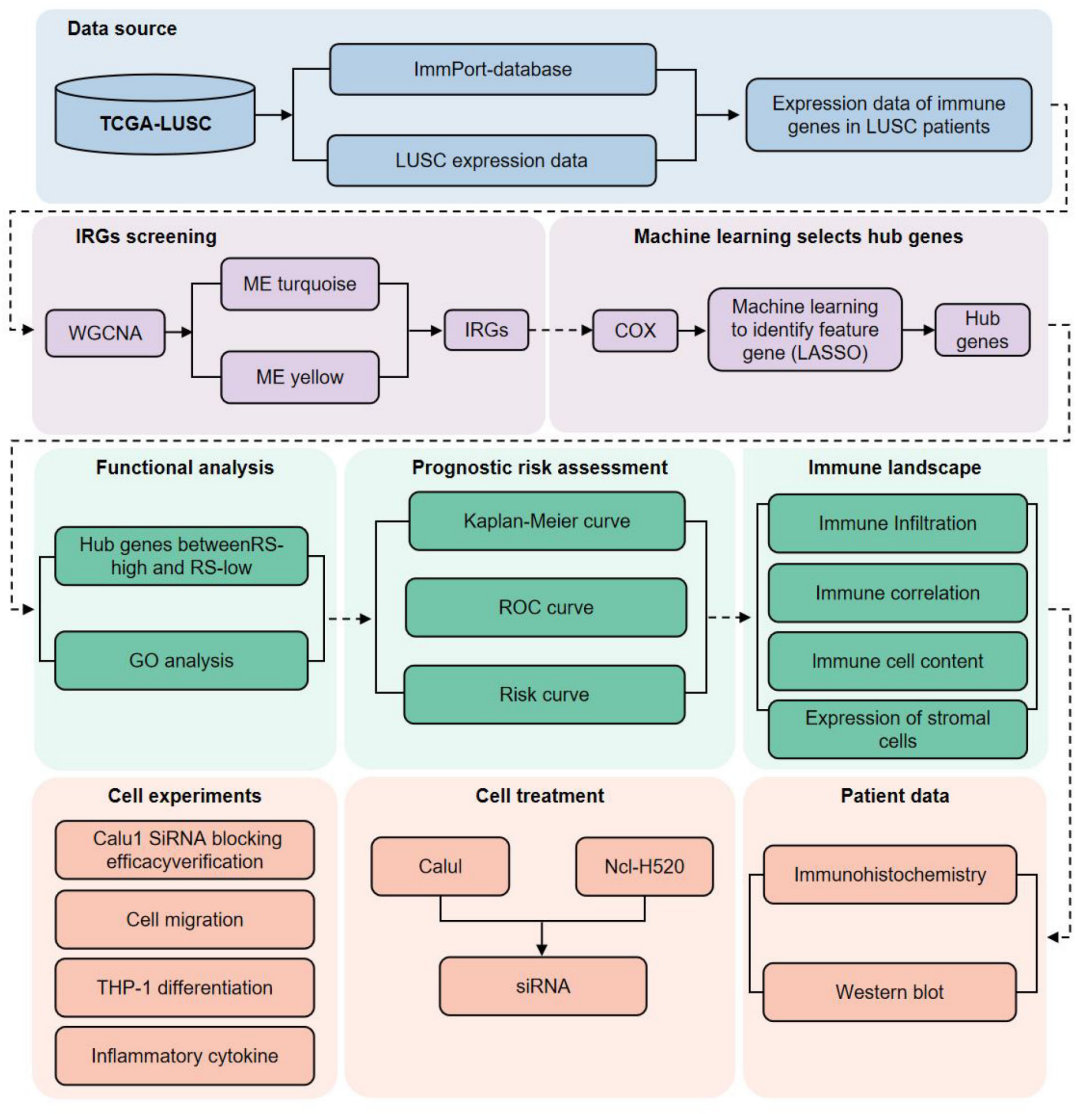
Flowchart of the study illustrating the construction and analysis of the immune-related gene (IRG) prognostic signature for LUSC.

All statistical analyses were conducted using R (version 4.0.1) and GraphPad Prism (version 9.5.0). Between-group comparisons for continuous variables were performed using the unpaired t-test (normal distribution) or Wilcoxon rank-sum test (non-normal distribution); comparisons among multiple groups used one-way ANOVA with Tukey’s *post-hoc* test. Categorical variables were analyzed with the χ² test. To account for multiple hypothesis testing across high-dimensional datasets (e.g., survival analysis of immune-related genes, differential expression, immune cell infiltration, and enrichment analyses), the Benjamini–Hochberg false discovery rate (FDR) correction was applied, with statistical significance set at FDR < 0.05. For analyses not involving mass parallel testing (e.g., Kaplan–Meier survival curves, ROC analysis), a two-sided P-value < 0.05 was considered significant. A power analysis confirmed that the sample size provided adequate statistical power to detect the expected effect sizes. The overall analytical workflow is illustrated in **Figure 1**.

## 3 Results

### 3.1 Construction of co-expression network and extraction of prognosis module

**Figure 2:**
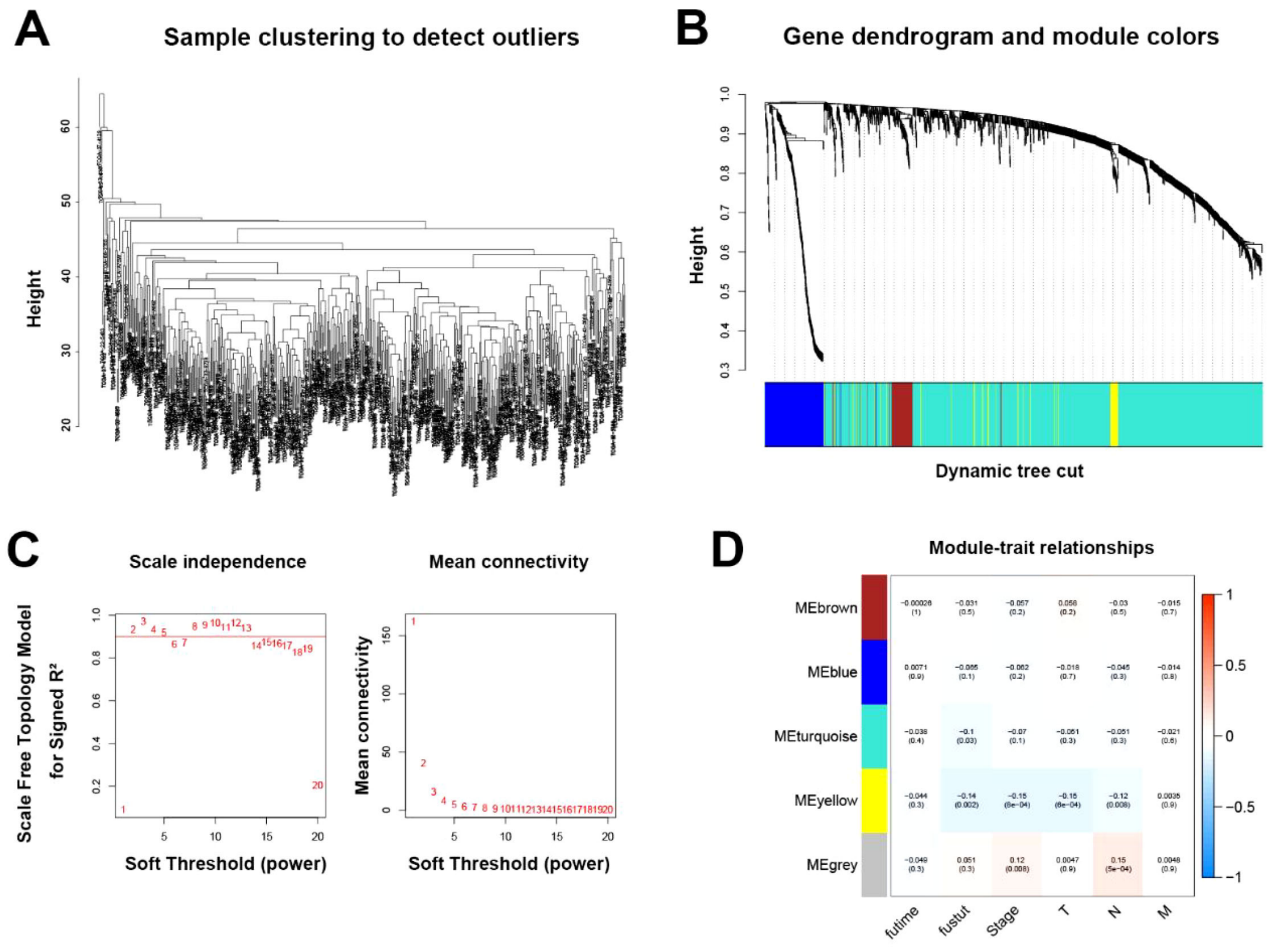
Identification of key modules based on WGCNA analysis. **(A)** Hierarchical clustering of 493 samples to detect potential outliers. **(B)** Gene dendrogram and module assignment using the dynamic tree cut method. The colored horizontal bar below the dendrogram represents the modules. **(C)** Selection of the soft-thresholding power based on scale-free topology model fit (left) and mean connectivity (right). **(D)** Correlations between each co-expression module and clinical features. The color indicates the strength of the correlation, and the number in parentheses shows the p-value for the correlation test. Significance levels indicated are based on Benjamini-Hochberg adjusted *P*-values (FDR).

After deleting abnormal outlier samples and normal samples, 493 LUSC samples remained (**Figure 2A**). Weighted correlation network analysis was performed to identify the genes most associated with LUSC prognosis (**Figure 2B**). When the soft threshold β is set to 2, the scale-free topological fitting index is greater than 0.8 (**Figure 2C**). The horizontal axis is the power of soft threshold, and the vertical axis represents the fitting index and mean connectivity of the scale-free topological model, respectively. After considering the stability of the average connectivity, β = 2 is selected. By building a co-expression network divided into 9 modules, a module clustering diagram (**Figure 2B**) was created. We selected modules related to survival status and survival time as prognosis among many modules, and selected meaningful modules. ME turquoise (P = 0.03) and ME yellow (P = 0.002) were the most meaningful modules, with correlation coefficients of -0.1 and -0.14, respectively (**Figure 2D**). Finally, after integrating the 50 genes in ME yellow and 1071 genes in ME turquoise (**Supplementary File 1**), we obtained the genes related to prognosis.

### 3.2 Construction of high-risk and low-risk prognostic classifier

**Figure 3:**
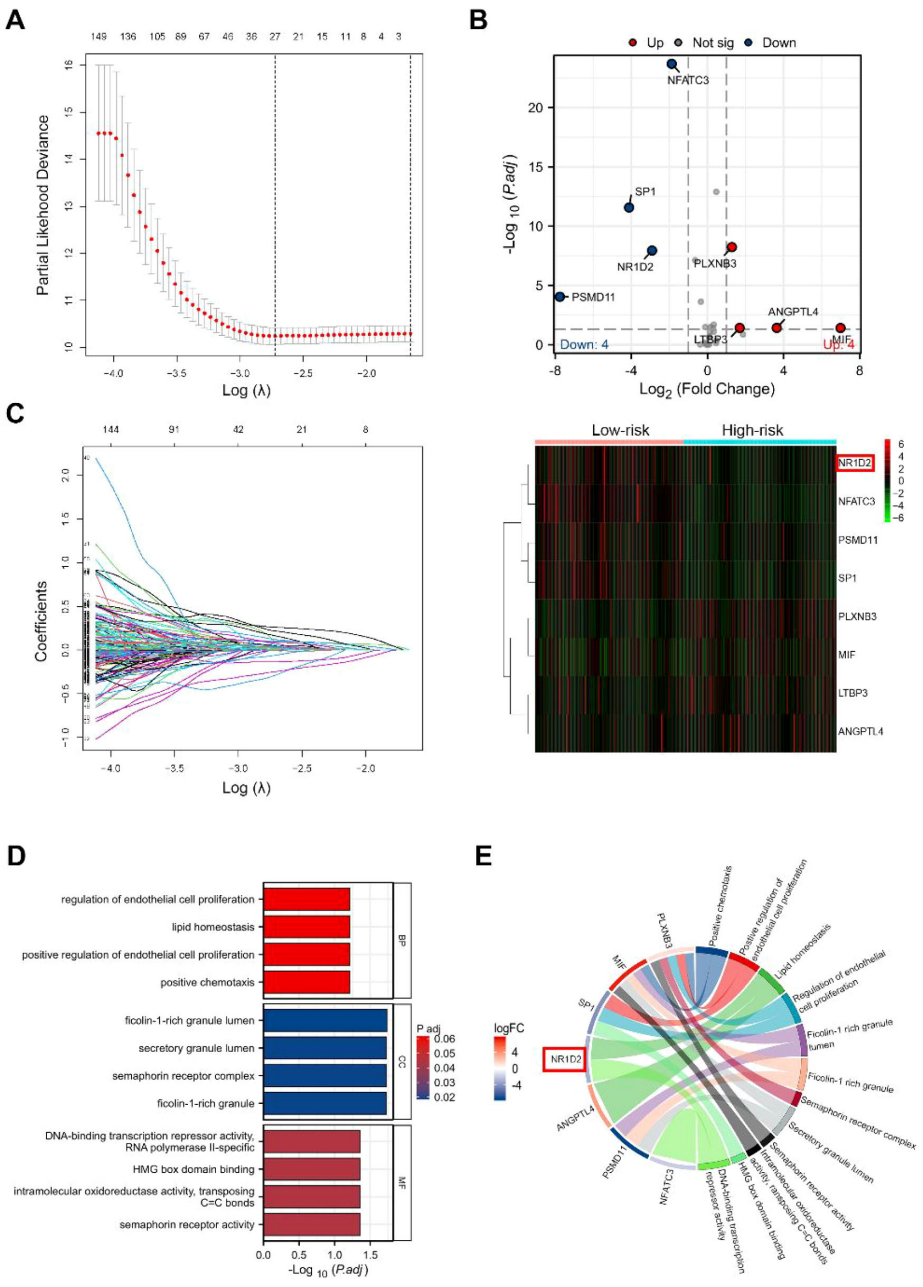
Machine learning-based screening of feature genes. **(A)** LASSO cross-validation plot showing the relationship between log (λ) and deviance, with dotted lines indicating optimal λ values. **(B)** Coefficient profiles for candidate genes as λ changes. **(C)** Differential expression genes (DEGs) between high-risk score and low-risk score groups. The volcano plot (left) highlights significantly upregulated and downregulated genes, while the heatmap (right) shows expression patterns of these DEGs across samples. **(D)** Gene Ontology enrichment analysis of the DEGs, illustrating top enriched biological processes. **(E)** Pathway analysis of the DEGs, visualized as a chord diagram linking genes to their corresponding pathways. Significance levels indicated are based on Benjamini-Hochberg adjusted *P*-values (FDR).

We performed univariate regression analysis of the prognostic genes described above. A total of 55 genes were obtained with p<0.05, correlation >0.4 (**Supplementary File 2**). We conducted Cox regression analysis to screen the most meaningful prognostic genes (**Figures 3A, B**). Eight genes *(PSMD1, ANGPTL4, LTBP3, MIF, NFATC3, NR1D2, PLXNB3, SP1*) were screened as the most meaningful genes, respectively (**Figure 3C**). According to the scoring formula, we calculated each sample and found the median value in order from largest to smallest. According to the median, we defined the samples with scores above the median as the high-RS group. In contrast, the lower group is defined as the low-RS group. Compared with the high-RS group, with the cutoff value adjusted p<0.05 and |log2FC|>1, the low-RS group has a higher expression of *PSMD1, NFATC3, SP1*, and *NR1D2* but has lower expression of *ANGPTL4* and *MIF* (p<0.05). In both the validation cohort and the training cohort, we found no difference in the expression of LTBP3 and PLXNB3 between the high-RS group and the low-RS group. Meanwhile, we conducted differential expression analysis between high and low-risk groups. Pathway and function enrichment analysis were conducted using by R language. Among the three levels of biological process (BP), cell component (CC) and molecular function (MF), 255 GO functions were enriched, and we visualized the top 4 GO pathways (**Figure 3D**). We visualized the molecules involved in the top four pathways in a statistically significant manner using a chord diagram (**Figure 3E**). The results showed that lipid homeostasis, regulation of endothelial cell proliferation, positive chemotaxis, positive regulation of endothelial cell proliferation significantly affected in the BP classification. In the MF classification, significant changes were observed in ficolin-1-rich granule lumen, ficolin-1-rich granule, semaphorin receptor complex, and secretory granule lumen. In CC classification, notable changes were found in intramolecular oxidoreductase activity, transposing C=C bonds, HMG box domain binding, DNA-binding transcription repressor activity, RNA polymerase II-specific, DNA-binding transcription repressor activity, and histone acetyltransferase binding.

### 3.3 Survival risk prediction of prognostic classifier

**Figure 4:**
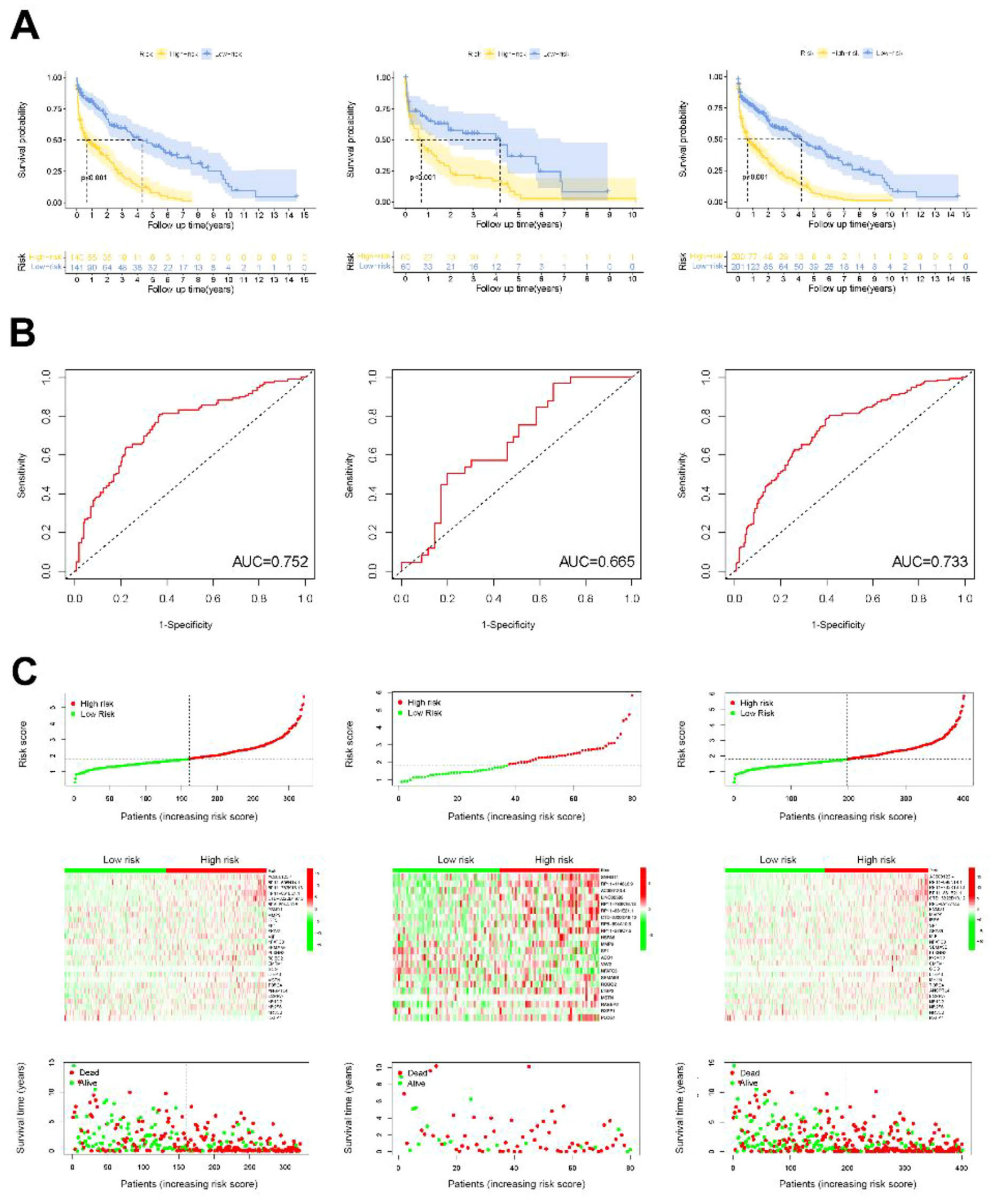
Validation of LUSC’s prognostic risk scoring model. **(A)** Kaplan-Meier survival curves for. patients stratified into high-risk and low-risk groups, with shaded areas indicating 95% confidence intervals. **(B)** ROC curves demonstrating the predictive performance of the risk model at different time points; the AUC values indicate its accuracy. **(C)** Risk plots showing the distribution of individual patient risk scores (top), corresponding survival status (middle) and a heatmap of key gene expression (bottom).

Through survival analysis, we found that low-RS was associated with significantly longer survival time (p <0.001), whereas high-RS (p <0.001) was associated with shorter survival time in patients with LUSC (**Figure 4A**). The AUC of the training group, validation group, and all testing groups were 0.752, 0.665, and 0.733, respectively, for the entire sample (**Figure 4B**). The time-dependent ROC curve shows that the classifier has strong prediction ability for the training data set. At the same time, we found that the low-RS group was associated with significantly better prognosis (p <0.001), whereas the high group (p <0.001) was associated with poor prognosis in patients with LUSC (**Figure 4C**).

### 3.4 Landscape of immune cell infiltration between high- and low-risk LUSC groups

**Figure 5:**
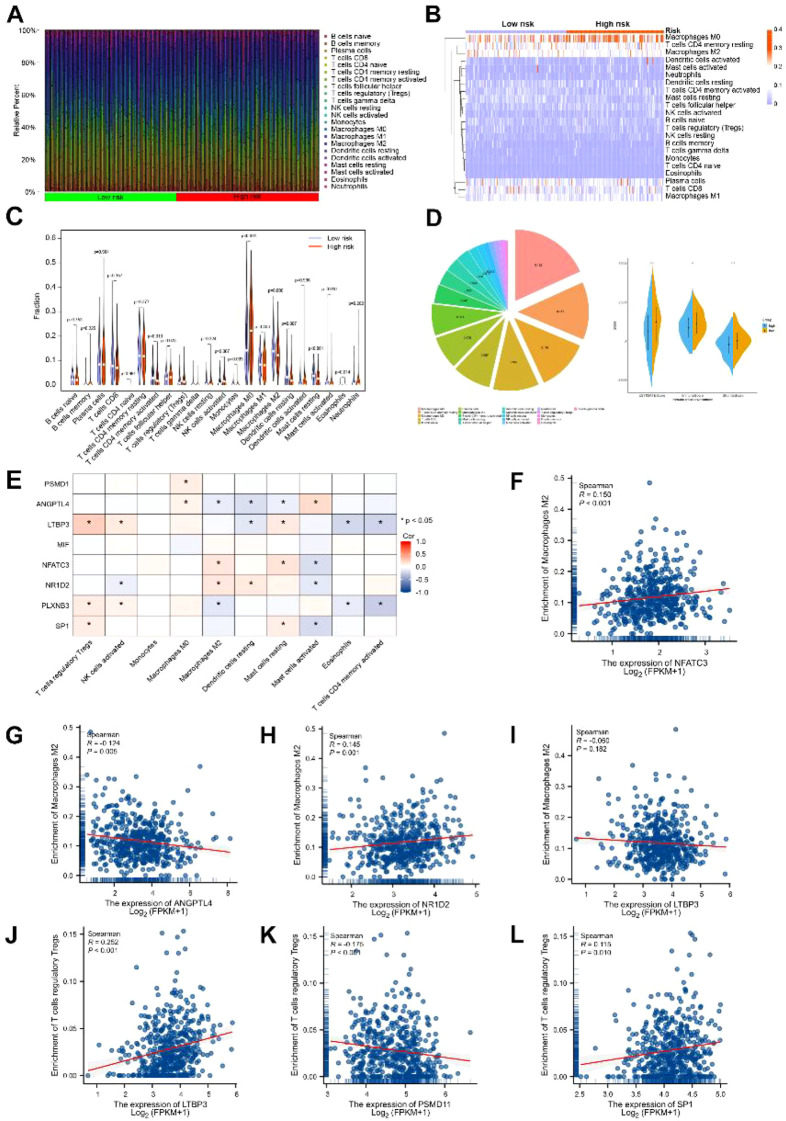
Results of immune cell infiltration between high- and low-risk groups. **(A, B)** Histogram of immune cell infiltration. **(C)** Box plot of differential expression of 22 immune cells. **(D)** Proportion of 22 types of immune cells in LUSC patients. **(E)** Comparison of stromal cells in high-risk and low-risk groups. **(F–L)** The correlation between characteristic genes and immune cells. Significance levels indicated are based on Benjamini-Hochberg adjusted *P*-values (FDR). (*p < 0.05).

The 22 immune cell infiltrations of LUSC are shown in **Figures 5A** and **Figure 5B**. Comparative analysis revealed significant differences in the infiltration abundances of several immune cell types, including Macrophages M2 and Tregs, between the high- and low-risk groups (all FDR < 0.05; **Figure 5C**). Macrophages M0, T cell CD4 memory resting, Macrophages M2, and T cells CD8 accounted for a large proportion of LUSC immune cell infiltration (**Figure 5D**). Moreover, we estimated the tumor microenvironment and we found that compared with the high-RS group, low-RS group had higher immune cell and stromal cell infiltrations (**Figure 5E**). Besides, we identified the correlation of biomarker genes with immune cells (**Figures 5F–L**). We selected M2 and Treg cells for a detailed correlation analysis. Our findings indicate that *LTBP3, PLXNB3*, and SP1 are positively correlated with Treg cells. Regarding M2 cells, we observed correlations with *ANGPTL4*, *NFATC3*, *NR1D2*, and *PLXNB3*. Specifically, *ANGPTL4* and *PLXNB3* are negatively correlated, whereas *NFATC3* and *NR1D2* are positively correlated with M2 cells.

### 3.5 *NR1D2* suppressed LUSC cell migration and invasion *in vitro*

**Figure 6:**
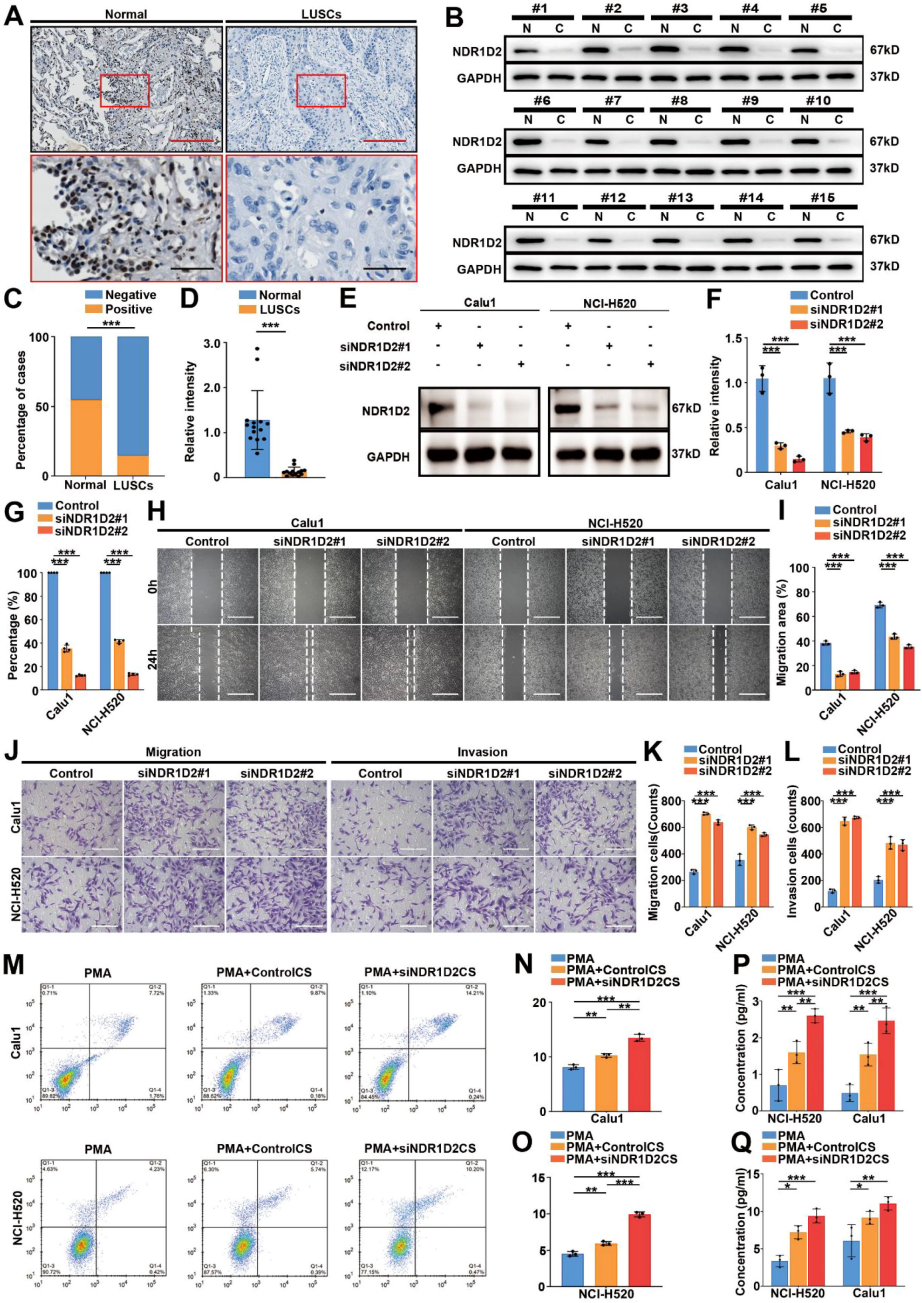
*NR1D2* is downregulated in LUSC, inhibits tumor cell migration, invasion, and modulates the immune microenvironment. **(A–D)** Immunohistochemistry **(A, C)** and Western blot **(B, D)** analysis show significantly reduced *NR1D2* expression in LUSC tissues compared to adjacent normal lung tissues (P < 0.001). Representative images **(A, B)** and quantification **(C, D)** are shown. **(E–G)** Efficient knockdown of *NR1D2* in Calu1 and NCI-H520 cells using two independent siRNAs (si*NR1D2*#1 and si*NR1D2*#2) confirmed by Western blot **(E, F)** and qPCR **(G)** (P <0.001 vs. siCtrl). **(H, I)** Wound healing assays demonstrate significantly impaired migratory capacity in *NR1D2*-silenced Calu1 and NCI-H520 cells. Representative images **(H)** at 0h and 24h and quantification of wound closure **(I)** are shown (P < 0.001 vs. siCtrl). **(J–L)** Transwell assays showing significantly reduced migration and invasion of Calu1 and NCI-H520 cell lines following *NR1D2* silencing. Representative images **(J)** and quantification of migrated/invaded cells **(K, L)** are presented (P < 0.001 vs. siCtrl). **(M–O)** Flow cytometry analysis of THP-1-derived macrophages cultured in conditioned medium (CM) from *NR1D2*-silenced tumor cells. Enhanced polarization towards CD206+CD163+ M2-like macrophages was observed. Representative dot plots **(M)** and quantification of CD206^+^CD163^+^ macrophage following exposure to CM from *NR1D2*-depleted Calu1 cells **(N)** and NCI-H520 cells **(O)** subsets are shown (**P < 0.001 vs. CM from siCtrl cells). **(P, Q)** ELISA assays show increased secretion of IL-10 **(P)** and TGF-β1 **(Q)** in macrophage supernatants following exposure to CM from *NR1D2*-depleted tumor cells (*P < 0.05, **P < 0.01, ***P <0.001 vs. CM from siCtrl cells). All data are presented as mean ± SD. Scale bars are provided in the respective image panels.

To investigate the functional role of *NR1D2* in LUSC, we first assessed its expression disparities in normal lung epithelial cells and LUSC tissue samples. Immunohistochemical analysis revealed significantly attenuated *NR1D2-*positive staining in LUSC tissues compared to adjacent normal lung tissues (P< 0.001, **Figures 6A, C**). Western blot analysis of 15 paired patient samples further confirmed the down-regulation of *NR1D2* in tumor tissues relative to adjacent normal tissues, with consistent GAPDH expression validating experimental reliability (P < 0.001, **Figures 6B, D**).

To establish functional relevance, we validated efficient *NR1D2* knockdown in Calu1 and NCI-H520 cell lines using siRNA transfection (s*iNR1D2*#1 and si*NR1D2*#2). Western blot analysis showing significant protein reduction (P<0.001, **Figures 6E, F**), and qPCR analysis showing significant mRNA reduction (P < 0.001, **Figure 6G**). Then, functional consequences were examined: (1) wound healing assays demonstrated markedly impaired migration upon *NR1D2* silencing (P < 0.001, **Figures 6H, I**). (2) Transwell migration and invasion assays exhibited significant elimination across Calu1 and NCI-H520 cell lines (P < 0.001, **Figures 6J–L**).

To evaluate immune modulation, flow cytometry analysis of THP-1 cells exposed to conditioned media from *NR1D2*-depleted tumors showed enhanced CD206^+^CD163^+^ macrophage polarization (P < 0.001, **Figures 6M–O**). ELISA analysis demonstrated elevated IL-10 and TGF-β1 secretion in THP-1 cells (P < 0.05, **Figures 6P, Q**). These data collectively indicate that *NR1D2* downregulation in LUSC suppresses metastatic behavior and fosters an immunosuppressive microenvironment.

## 4 Discussion

According to the World Health Organization’s International Agency for Research on Cancer (IARC), lung Cancer is the leading cause of cancer-related deaths worldwide, 2.26 million people were diagnosed with lung cancer, and 1.8 million people died as a result of the disease 2020 (https://gco.iarc.fr/today, accessed February 2021).

In this study, IRGs in TCGA were extracted for WGCNA analysis, identifying the prognostic-related modules, applying the bioinformation methods such as built-in functions in the R package, cox proportional hazard regression model, ROC analysis, and lasso regression analysis to systematically analyze the RNA-seq data associated with lung squamous cell carcinoma. 8 IRGs were screened out: *PSMD1, ANGPTL4, LTBP3, MIF, NFATC3, NR1D2, PLXNB3, SP1*(**Figure 3C**). Although some IRGs in our results have not been functionally annotated and clearly researched, we investigated other biomarkers used in our classifier. We found that most IRGs play a critical role in the aspects of cancer cell metastasis, migration, and angiogenesis. Sequencing found that the expression level of *PSMD1* was increased in lung cancer tissues, which was the most significant difference among all genes. Meanwhile, research has shown that *PSMD1* is overexpressed in lung adenocarcinoma. Overexpression of *PSMD1* can decrease the levels of cleaved caspase-3 and Bax while increasing the level of Bcl-2. *PSMD1* plays a crucial role in regulating cancer occurrence and progression (12, 13). Angiopoietin-like 4 (*ANGPTL4*) is a multifunctional secretory protein (14). Recent research has emphasized its significant role in cancer, including promoting cell migration, proliferation, and glycolysis (15). Elevated expression of *ANGPTL4* has been observed in lung adenocarcinoma, and its expression is significantly correlated with poor prognosis (16). In our prognostic model, *ANGPTL4* emerged as a potential biomarker (**Figures 3A–C**). Literature indicates that Latent TGF-β Binding Protein 3 (LTBP-3) plays a crucial role in cancer metastasis by regulating angiogenesis and influencing the early dissemination and invasion of cancer cells from the primary tumor, although it is not essential for later stages of metastasis, such as vascular arrest, extravasation, and colonization (17). Macrophage Migration Inhibitory Factor (MIF) is a pro-inflammatory and pro-tumorigenic cytokine secreted by various immune cells, including monocytes, macrophages, neutrophils, T cells, B cells, dendritic cells, and eosinophils, as well as by certain endocrine, endothelial, and epithelial cells under inflammatory stimuli or injury (18). Studies have shown that MIF interacts with CD74 or CXCR2 and CXCR4 receptors, activating signaling pathways such as protein kinase B (AKT), extracellular signal-regulated kinases (ERK), and nuclear factor kappa-B (NF-κB), thereby promoting tumor proliferation, angiogenesis, and metastasis (19, 20). The *NR1D2* gene encodes a protein known as Rev-erbβ, a member of the nuclear receptor superfamily, which plays a crucial role in regulating circadian rhythms and metabolism. NFATc3 (also known as NFAT4) is a transcription factor belonging to the Nuclear Factor of Activated T-cells (NFAT) family (21). Previous research has shown that a deficiency in NFATc3 can attenuate the inflammatory response induced by hypothalamic injury (22). NFATc3 is also important in M1 macrophage polarization and adipose tissue inflammation (23). Furthermore, NFATc3 has been shown to enhance the proliferation and invasive capabilities of colorectal cancer (CRC) cell lines (24). Plexin B3 (PLXNB3), a large transmembrane protein, interacts with semaphorins to mediate axon guidance during the development of the central nervous system. Recent analysis of pan-cancer data from The Cancer Genome Atlas (TCGA) revealed that intratumoral expression of PLXNB3 is associated with reduced patient survival (25). Specificity protein 1 (SP1), the first mammalian transcription factor to be cloned and characterized, can bind to DNA through its binding domain to activate or repress the transcription of various target genes, such as cyclin D, E-cadherin, FLIP, TGF-β, and HDAC, regulating processes like cell cycle, angiogenesis, apoptosis, tumorigenesis, and chromatin remodeling (26). Extensive literature reports that SP1 is overexpressed in approximately 90% of malignant tumors, particularly in cancer cells resistant to radio- and chemotherapy (27, 28). Elevated expression of SP1 promotes tumor progression by regulating cell proliferation, migration, and invasion, and affects the resistance of tumor cells to radio- and chemotherapy. Although research on the role of these genes in the TME or in cancer remains insufficient, all these genes above may become new prognostic biomarkers for LUSC. The area under the ROC curve is greater than 0.5, and the survival curves indicate that the IRGs classifier has high predictive value and accuracy (29).

Compared with targeted therapy and surgical treatment, the proportion of patients treated with immunotherapy grew fastest (the annual growth rate of 21 years was 49.2%) among patients with lung cancer, which greatly improved the 5-year survival rate of patients with advanced-stage lung cancer. In this study, we applied the immune cell scoring and estimated the score of immune cells and stromal cells. Furthermore, we used immune infiltration analysis to evaluate the infiltration of immune cells in lung cancer. We found that immune-related cells infiltrated more in the low-RS group than in the high-RS group. In addition, we found that Tregs, NK cells were activated, and macrophages M0 significantly increased in high-RS group (**Figure 5C**). Monocytes, CD4^+^ memory activated, Dendritic cells resting, macrophages M2, and Mast cells resting decreased in the high-RS group (P < 0.05). Statistics showed that these cells account for the vast majority of immune cells in cancer patients, which play an important role in the immune microenvironment. M2 macrophages can promote the expression of anti-inflammatory cytokines such as IL-10, prostaglandin E2, TGF-β, MMP, and VEGF, inhibit dendritic cells and CD8^+^ T lymphocytes, inhibit immune response, and promote tumor growth and metastasis (30). It was interesting that macrophage M2 was lower in the high-RS group compared with the low-RS group. Studies have shown that activated memory CD4 T cells and mast cells are significantly correlated with prognosis, and the abundance of mast cells is significantly correlated with TNM staging (31). Bruno et al. found that tumor-infiltrating B cells could present endogenous tumor antigens to CD4^+^ TIL and change the phenotype of CD4^+^ TIL *in vitro*, and the activated tumor-infiltrating B cells were related to the response of activated IFN-γ ^+^ CD4 ^+^ T cells (32). Natural killer (NK) cells can directly recognize and kill tumor cells, which play a key role in anti-tumor immunity. Although tumor cells form a variety of strategies to avoid CD8^+^ T cell recognition, they are also attacked by NK cells (33). NK cells combined with Pembrolizumab can improve the survival rate of patients with advanced non-small cell lung cancer (34). M2 macrophages can promote the expression of anti-inflammatory cytokines such as IL-10, prostaglandin E2, TGF-β, MMP, and VEGF, inhibit dendritic cells and CD8^+^ T lymphocytes, inhibit immune response, and promote tumor growth and metastasis (30). Tumor microenvironment is composed of non-hematopoietic stromal cells, extracellular matrix, lymphocytes, and bone marrow cell subsets (35). Under normal physiological conditions, the dynamic balance between tissues can prevent the occurrence and development of tumors. Under pathological conditions, the interaction between the tumor and its microenvironment can promote the occurrence, growth, and metastasis of tumors. Immune cells in the tumor microenvironment are new targets for immunotherapy of malignant tumors, and the tumor microenvironment plays an important role in the formation, growth, and metastasis of immune cells. In this study, we estimated the score of immune microenvironments and found that low-RS group enjoyed higher immune score compared with high-RS group (**Figure 5E**). Then we screened out the IRGs correction with immune cells. We found all IRGs related to Tregs, NK cells, Macrophages M2, and their phases. In the immune microenvironment of lung cancer, the localization of NK cells in human and mouse lung cancer is similar, mainly located at the edge of invasion around the tumor lesions, and rarely directly contact with cancer cells (36, 37). NK cells exert anti-tumor characteristics mainly by expressing Fc receptors and releasing extracellular vesicles. M2-polarized macrophages are usually called tumor-associated macrophages (TAMs), which play a promoting role in tumor growth, migration, and invasion. At present, emerging studies have shown that TAM infiltration in tumor sites is often related to the poor prognosis of various cancers (38). At the same time, M2 macrophages accounted for the top three in lung cancer, reaching 11.8%. In conclusion, NK cells and M2 macrophages may have some potential effects. In recent years, the roles and interactions of Th17 and Treg cells, two major subsets of CD4^+^ T lymphocytes, have garnered significant attention in the context of lung cancer, particularly non-small cell lung cancer (NSCLC). Clinical studies have found that the numbers of Th17 and Treg cells, as well as the Th17/Treg ratio, are elevated in the peripheral blood and bronchoalveolar lavage fluid (BALF) of NSCLC patients compared to healthy individuals. Moreover, the quantities of Th17 and Treg cells in peripheral blood are closely related to clinical stage, tumor burden, chemotherapy sensitivity, and prognosis (39, 40).An increase in Treg cell numbers or their enhanced function can lead to upregulation of immune checkpoints such as cytotoxic T-lymphocyte-associated protein 4 (CTLA-4), glucocorticoid-induced tumor necrosis factor receptor (GITR), and programmed cell death protein 1 (PD-1). This upregulation can subsequently modulate the secretion of TGF-β, IL-6, IL-10, and IL-17, thereby inhibiting the production and function of CD4^+^/CD8^+^ T cells, dendritic cells (DCs), and natural killer (NK) cells. Consequently, this promotes immune evasion by tumor cells (i.e., tumor immune tolerance) and facilitates the metastasis and progression of lung cancer (41, 42).PD-1 contributes to the persistence of PD-1^+^TIM-3^+^ T cells by binding with TIM-3 ligand galectin-9 (Gal-9), and mitigates Gal-9/TIM-3-induced cell death. Anti-Gal-9 therapy selectively expands TIM-3^+^ cytotoxic CD8 T cells and immunosuppressive regulatory T cells (Tregs) within tumors. The combination of anti-Gal-9 and agonistic antibodies targeting the co-stimulatory receptor GITR can deplete Tregs, thereby inducing synergistic anti-tumor activity (43).

Several limitations should be acknowledged. First, our study relies on a single-cohort design, which restricts the generalizability of the findings. The absence of independent external validation cohorts further limits the robustness of the eight-gene signature. Future large-scale, multi-center prospective studies will be conducted to further confirm its prognostic performance. Second, inconsistencies were observed between our bioinformatics analysis and experimental findings. While bioinformatics results suggested a positive correlation between *NR1D2* expression and M2 macrophage infiltration, likely reflecting complex microenvironmental co-regulation *in vivo*; cellular experiments showed that *NR1D2* downregulation promotes CD206^+^CD163^+^ M2 polarization, indicating that *NR1D2* may function as a negative regulator of M2 differentiation under *in vitro* conditions. These context-dependent differences highlight the multifaceted role of *NR1D2* in shaping the immune landscape of LUSC. Additional mechanistic studies, both *in vitro* and *in vivo*, are required to elucidate the biological functions of *NR1D2*. Third, although the 8-IRG signature shows potential for clinical translation and could theoretically be adapted to liquid biopsy platforms for minimally invasive monitoring, its ability to predict immunotherapy response remains unverified. In the TCGA cohort, few LUSC patients had complete follow-up and immunotherapy outcome data, which is insufficient for reliable evaluation. Prospective cohorts with well-annotated treatment information will be essential to validate this aspect. Finally, translating the signature into a clinically feasible test remains challenging. Developing a standardized, clinically feasible assay and validating its predictive utility in prospective interventional trials will be essential steps toward determining whether the signature can reliably guide immunotherapy decisions.

## 5 Conclusion

In this study, for the first time, we used the immune gene classifier to divide the samples into high-RS and low-RS groups, and then analyzed the differences in the immune environment between the two groups, as well as the differential expression of genes, so as to identify potential therapeutic drugs. *NR1D2* is downregulated in LUSC and promotes tumor progression through macrophage polarization and enhanced migration. These results show that a parsimonious IRG signature can serve as a tool for predicting LUSC prognosis and inform personalized immunotherapeutic strategies.

## Data Availability

The datasets presented in this study can be found in online repositories. The names of the repository/repositories and accession number(s) can be found in the article/**Supplementary Material**.

## Supplementary material

Supplementary Figure 1 Correlation between characteristic genes and macrophage M2 infiltration.

Supplementary Figure 2 The quantification of CCK8 assay in Calu1 **(A)** and NCI-H520 **(B)** cell lines from 24h to 96h, after *NR1D2* knockdown using two independent siRNAs (si*NR1D2*#1 and si*NR1D2*#2).

Supplementary File 1 List of genes in ME yellow and in ME turquoise.

Supplementary File 2 List of prognostic genes.
