# Supplementary figures and images for: Identification of immune-related prognostic biomarkers in lung squamous cell carcinoma microenvironment
Source: Front Immunol. 2026 Jan 5;16:1724319. doi: 10.3389/fimmu.2025.1724319 (PMC12813068; doi:10.3389/fimmu.2025.1724319)

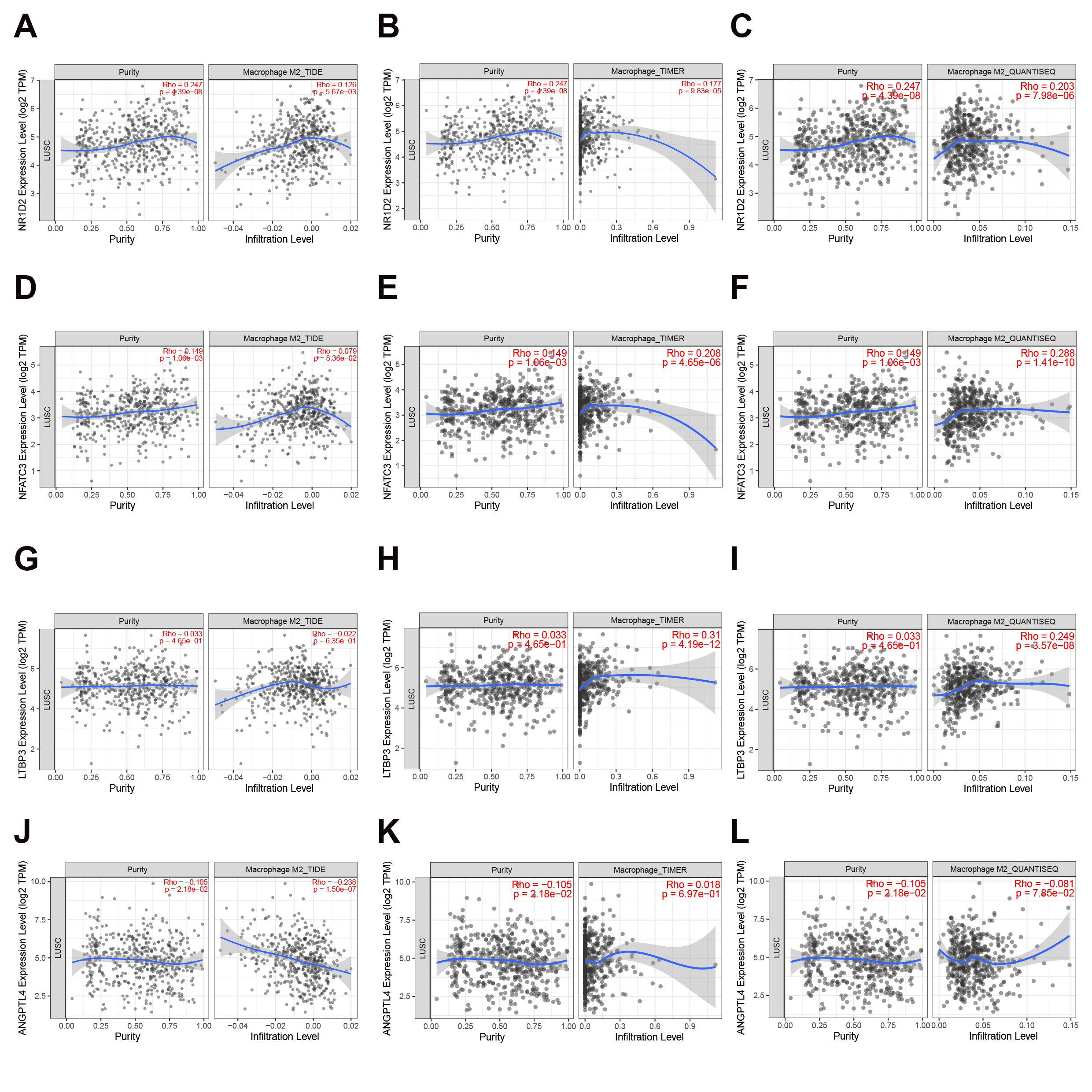

Supplement: Supplementary Figure 1 — Correlation between characteristic genes and macrophage M2 infiltration. [file Image1.tif]

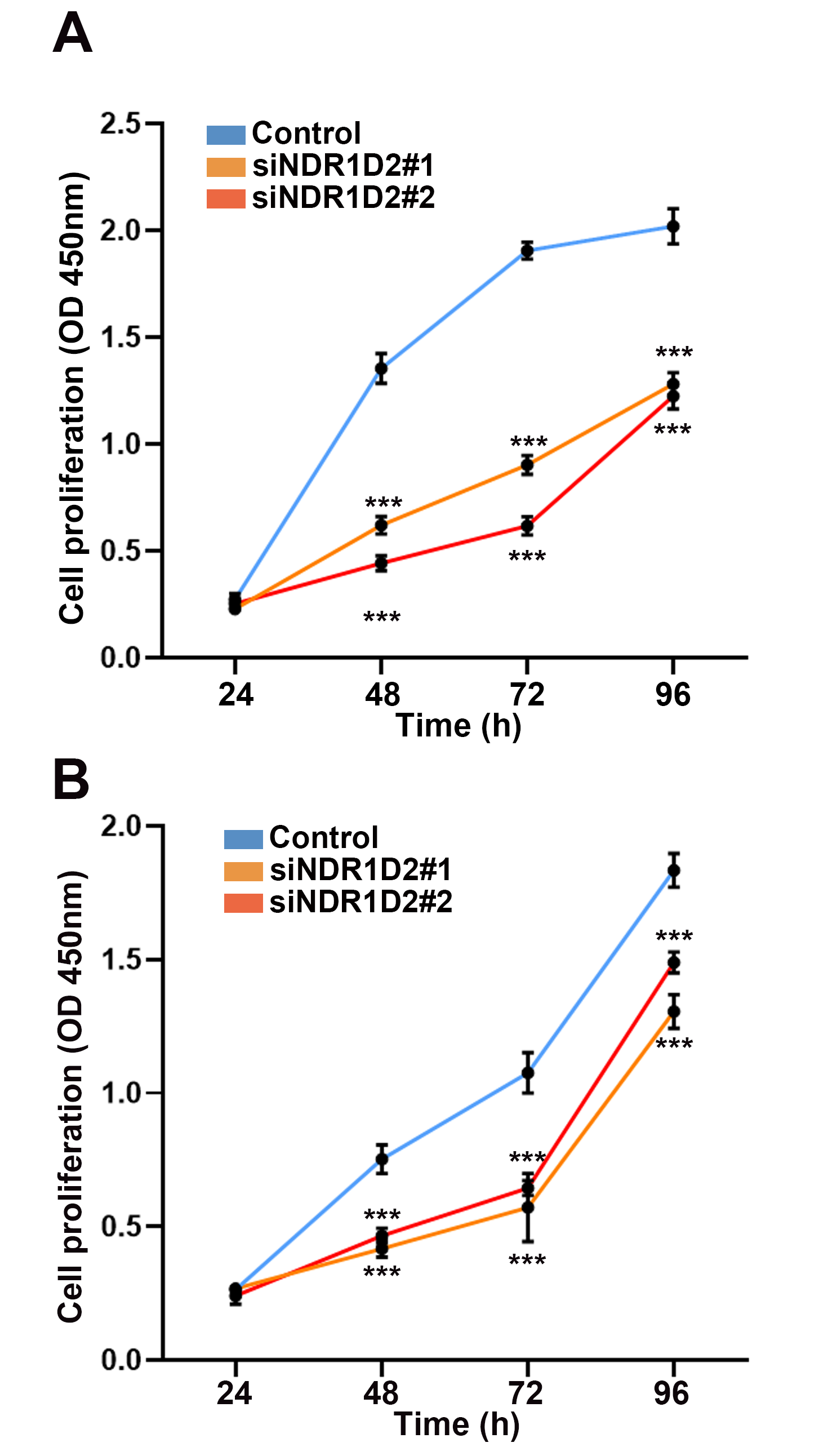

Supplement: Supplementary Figure 2 — The quantification of CCK8 assay in Calu1 (A) and NCI-H520 (B) cell lines from 24h to 96h, after NR1D2 knockdown using two independent siRNAs (siNR1D2#1 and siNR1D2#2). [file Image2.tif]
